# Development of Molecular Magnetic Resonance Imaging Tools for Risk Stratification of Carotid Atherosclerotic Disease Using Dual-Targeted Microparticles of Iron Oxide

**DOI:** 10.1007/s12975-021-00931-3

**Published:** 2021-07-25

**Authors:** Joyce M. S. Chan, Park Sung Jin, Michael Ng, Joanne Garnell, Chan Wan Ying, Chong Tze Tec, Kishore Bhakoo

**Affiliations:** 1 Translational Cardiovascular Imaging Group, Institute of Bioengineering and Bioimaging (IBB), Agency for Science, Technology and Research (A*STAR), Singapore, Singapore; 2 Department of Vascular Surgery, Singapore General Hospital, SingHealth, Singapore, Singapore; 3 Division of Oncologic Imaging, National Cancer Centre, SingHealth, Singapore, Singapore; 4 Institute of Bioengineering and Bioimaging (IBB), Agency for Science, Technology and Research (A*STAR), Singapore, Singapore

**Keywords:** Stroke, Atherosclerosis, Vulnerable carotid plaques, Inflammation, Iron oxide particles, Risk stratification

## Abstract

Identification of patients with high-risk asymptomatic carotid plaques remains a challenging but crucial step in stroke prevention. Inflammation is the key factor that drives plaque instability. Currently, there is no imaging tool in routine clinical practice to assess the inflammatory status within atherosclerotic plaques. We have developed a molecular magnetic resonance imaging (MRI) tool to quantitatively report the inflammatory activity in atherosclerosis using dual-targeted microparticles of iron oxide (DT-MPIO) against P-selectin and VCAM-1 as a smart MRI probe. A periarterial cuff was used to generate plaques with varying degree of phenotypes, inflammation and risk levels at specific locations along the same single carotid artery in an Apolipoprotein-E-deficient mouse model. Using this platform, we demonstrated that in vivo DT-MPIO-enhanced MRI can (i) target high-risk vulnerable plaques, (ii) differentiate the heterogeneity (i.e. high vs intermediate vs low-risk plaques) within the asymptomatic plaque population and (iii) quantitatively report the inflammatory activity of local plaques in carotid artery. This novel molecular MRI tool may allow characterisation of plaque vulnerability and quantitative reporting of inflammatory status in atherosclerosis. This would permit accurate risk stratification by identifying high-risk asymptomatic individual patients for prophylactic carotid intervention, expediting early stroke prevention and paving the way for personalised management of carotid atherosclerotic disease.

**Supplementary Information:**

The online version contains supplementary material available at 10.1007/s12975-021-00931-3.

## Introduction

Stroke is the second leading cause of death and long-term disability globally [1]. Atherosclerosis, defined by persistent inflammation and accumulation of lipid-rich plaques in arterial walls [2], is the principal cause of stroke. Ischaemic stroke contributes to more than 80% of all strokes [1], whereby 15 to 30% of these are caused by thromboembolic events arising from carotid atherosclerotic plaques [3]. The conventional procedures for stroke prevention in symptomatic carotid disease are carotid endarterectomy and carotid artery stenting. For asymptomatic disease, however, surgery or stenting become increasingly debatable with more effective medical therapies, and with a decreased annual stroke rate to about 1%, justification for carotid intervention is more difficult [3, 4]. Nevertheless, asymptomatic stable atherosclerotic plaques can become acutely unstable and lead to thromboembolic stroke, but currently there is no reliable imaging tool to discriminate between stable and vulnerable asymptomatic plaques.

The advancements in molecular imaging techniques have helped to shift the emphasis from imaging plaque structure alone, to directly reporting the pathophysiological activities of atherosclerosis at the molecular and cellular levels [5]. Molecular MRI aims to concurrently image the precise anatomy and biological processes in vivo by acquiring different contrast weightings, enabling co-registration of molecular with anatomical information into a single imaging modality [6]. Iron oxide-based contrast agents are emerging as favoured MR contrast because they are relatively easy to synthesise with a known safety profile and potent signal [6]. Iron oxide contrast agents augment sensitivity and enhance diagnosis by reducing T2 and T2* relaxation times to generate hypointense (dark) signals on T2- and T2*-weighted MRI [6].

Molecular MRI encompasses ‘passive’ and ‘active’ targeting strategies. ‘Passive’ targeting strategy, for example utilising the non-specific uptake of ultrasmall superparamagnetic particles of iron oxide (USPIO) by plaque macrophages as surrogate markers of intraplaque inflammation, has been reported in both animals [7] and human [8]. ‘Active’ targeting strategy involves binding selectively to the molecular targets. Larger microparticles of iron oxide (MPIO) have been used in imaging vascular targets [9–11]. With higher iron oxide content, MPIO augments MR signal to enhance direct reporting of molecular targets in vivo. ‘Active’ molecular imaging can be attained through conjugating MPIO to specific targeting antibodies against inflammatory antigens expressed by ‘active’ vascular endothelium [9–11].

Inflammation is a key factor to drive plaque destabilisation with chronic benign plaques converting into acutely unstable ones, leading to clinical sequelae of thromboembolism. The over-expression of adhesion molecules, such as vascular cell adhesion molecule 1 (VCAM-1 [cluster of differentiation (CD)106]) and P-selectin (CD62P), on the activated endothelium mediates monocyte recruitment into the vascular tissues [12]. The coaction of rolling and firm adhesion by selectins and VCAM-1 was shown to produce synergistic effect on monocyte-endothelial binding [13]. VCAM-1 expression is also found on the other major plaque constituents, such as activated macrophages and smooth muscle cells [12]. Capitalising on the abundance and vital roles of these inducible adhesion molecules, dual-targeted iron oxide particles directed at both selectins and VCAM-1 was synthesised and shown to be more effective than either alone in our study on human carotid plaques [14] and previous studies [9, 11]. Furthermore, the tight spatio-temporal regulation of these adhesion molecules and the instant accessibility via the circulation render them imperative targets for functional molecular imaging and potential targeted therapeutics [12].

In this study, we have developed fluorescent-labelled dual-targeted MPIO (DT-MPIO) against P-selectin and VCAM-1 as a dual-imaging modality contrast agent for the following: (i) in vivo MR imaging of inflammation in atherosclerosis and (ii) ex vivo optical imaging for subsequent histological validation. This in vivo molecular imaging strategy was utilised to assess plaque inflammation in the carotid arteries of an Apolipoprotein E-deficient (ApoE^−/−^) mouse model. Furthermore, we sought to determine whether DT-MPIO enhanced MR imaging tool could (i) target and differentiate vulnerable carotid plaques from stable plaques; and (ii) quantitatively report the inflammatory status of plaques for risk stratification.

## Methods

### Animals

All animal experiments were performed in agreement with a protocol approved by the Institutional Animal Care and Use Committee for Biological Resource Center at A*STAR, Singapore (IACUC #191,459). ApoE^−/−^ (apolipoprotein E-deficient mice, ApoE knock-out mice (Taconic Biosciences)) were used to develop the cuff implanted atherosclerosis animal model.

### Cuff Implantation in Carotid Arteries

**Fig. 1:**
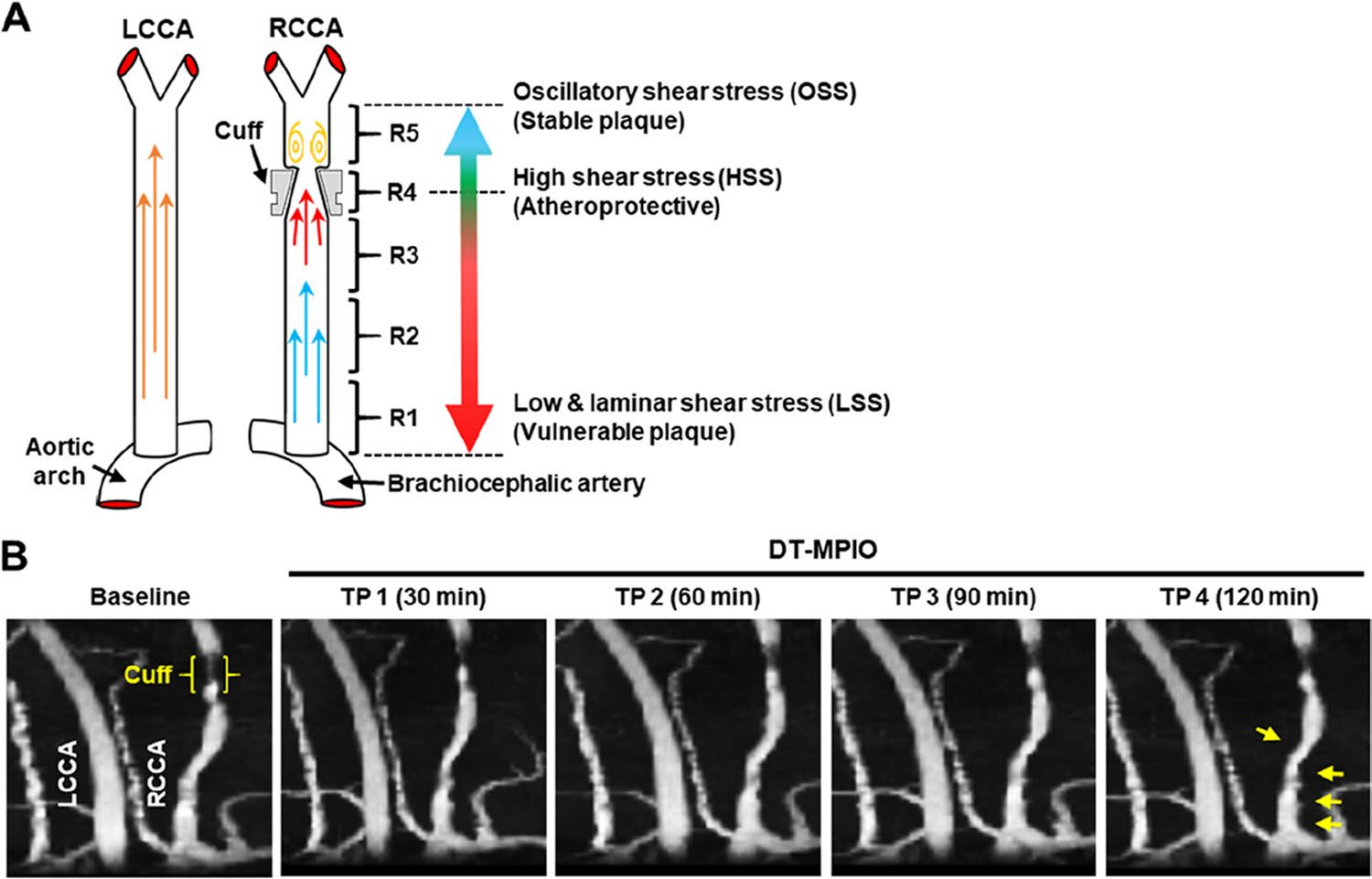
Targeting and differentiating vulnerable carotid plaques from stable plaques by in vivo DT-MPIO-enhanced MRI. (A) Schematic diagram of the implanted cuff. The periarterial cuff was implanted in right common carotid artery (RCCA) to induce unstable vulnerable plaques in the upstream (R1, R2 and R3), athero-protective region within the cuff (R4) and stable plaques in the downstream (R5). The left common carotid artery (LCCA) remained non-treated as control. (B) In vivo DT-MPIO-enhanced MRI. New areas of distinct hypointense signal were observed in R1–3 of RCCA from 30 min after DT-MPIO administration and remained evident throughout the imaging duration of 2 h. No new distinct hypointense signal was detected in R4, R5 and throughout LCCA in the post-contrast images

The shear-stress modifying cuff (Promolding BV) and surgical procedure were reported in earlier studies [9, 15, 16]. The cuff with a tapered end produced three distinctive regions: low shear stress (LSS) upstream of cuff placement, high shear stress (HSS) within cuff and oscillatory shear stress (OSS) downstream of the implanted carotid artery (Fig. 1A). It was generally accepted that LSS induces development of vulnerable, inflamed plaques, OSS promotes formation of stable plaques, whilst HSS protects against atherosclerosis [9, 15, 16]. ApoE^−/−^ mice (8 weeks old) were fed with a high-fat diet for 2 weeks preceding cuff implantation. At age 10 weeks, surgery was performed to implant the cuff on the right common carotid artery (RCCA), leaving the left common carotid artery (LCCA) untreated as control.

### In Vivo Magnetic Resonance Imaging of Carotid Arteries

ApoE^−/−^ mice were imaged at 30 weeks post-cuff implantation. In vivo MRI of carotid arteries was carried out on a 11.7 T MRI scanner (Bruker, BioSpec), using a 40-mm inner diameter transmit-receive volume coil (Bruker). Mice were kept under inhalation anaesthesia with 1.5–2% isoflurane. A 3-dimensional fast low-angle shot (FLASH) gradient-echo-based time-of-flight angiography (TOF-MRA) sequence was used to obtain a pre-contrast baseline scan: TR: 12.0 ms; TE: 2 ms; FOV: 24 × 24 × 10 mm; acquisition matrix: 256 × 256 × 64; average: 4; slab thickness: 10 mm; flip angle: 20°; acquisition time: 10m54s. Once baseline scans were completed, DT-MPIO (*n* = 20) or control IgG-MPIO (*n* = 6), 30 mg iron/kg body weight in 150 µL PBS, was administered through tail vein. Thereafter, MRA of carotid arteries was continuously acquired using the same sequence, up till 2 h post-contrast administration.

### Analysis of MR Images

The MR images were analysed using Dataviewer (Bruker) and CTan (Bruker) software. In Dataviewer, the image of each RCCA (cuff) and LCCA (control) was re-orientated into a cranio-caudal position. With the pre-contrast carotid as the reference image, the post-contrast carotid with greatest hypointense signal was registered to it. Using CTan, an interpolated region of interest (ROI) was first drawn throughout the 64 axial slices to include the carotid vessels only. Subsequently, a predefined threshold is selected to convert the gray scale images into binary images. The carotid area in each slice is calculated using the total number of white pixels in each slice and a plot of carotid area versus slice number is obtained. The magnitude of change in hypointense signal between the pre- and post-contrast carotid image, induced by MPIO, was quantified by the difference in area under the curve (AUC) between the pre- and post-contrast graph plots.

### Analysis of Histological Images

All samples were observed under the same conditions with a Ni-E, Ri2 microscope (Nikon®) for bright-field images, and a Ti fluorescent microscope (Nikon®) for fluorescent signals including DT-MPIO signal. Each sample was measured for fluorescent intensity and stained area using the NIS-Elements software (Nikon®). In each sectioned sample, the intima of the carotid artery (plaque area) was delineated as the region of interest (ROI) for analysis. The intensity of the tunica intima was measured as the average intensity per area (AU/µm^2^). The acquired values were used for the statistical analysis.

### Statistical Analysis

Sectioned samples from ApoE^−/−^ mice that were stained by H & E, Oil Red O and fluorescent IHC (MOMA, CD62P, VCAM-1, SMA) were analysed for the statistical results.

The box and whisker plot was used to represent the association between MR signal change and fluorescent signal in the different regions of the carotid (R1, 2, 3 and 5). The average values of DT-MPIO, Oil Red O and IHC signals (MOMA, CD62P, VCAM-1 and SMC) in each region of each mouse were represented. Student’s *t*-tests were calculated between regions R1, 2, 3 and 5 for statistical significance. False discovery rate (FDR) was used to correct for the multiple comparisons.

Furthermore, the correlations between MRI signal change and (i) the fluorescent signal of DT-MPIO, and (ii) expression of individual biomarkers (CD62P, VCAM-1, MOMA, Oil Red O and SMC) were examined. Samples from R1, 2, 3 and 5 were used. R4 has been well established as a plaque-free region and was excluded from the cohort correlation analysis. The regression analysis and *R*-square value were computed to assess the correlation between MRI signal change and of the expression of each biomarker. A value of *p* < 0.05 will be considered statistically significant.

Detailed analysis of synthesis of fluorescent-tagged dual antibody-conjugated MPIO and histological analysis are available in the Supplementary Methods.

## Results

### Targeting and Differentiating Vulnerable Carotid Plaques from Stable Plaques

#### In Vivo DT-MPIO-Enhanced MRI

In vivo MR angiography (MRA) of carotid arteries was carried out to determine if DT-MPIO can target and discriminate the high-risk vulnerable plaques from the low-risk stable ones. In the DT-MPIO group, new areas of distinct hypointense signal were observed on the luminal side in LSS region (R1-3) of RCCA from 30 min after DT-MPIO administration and remained evident throughout the imaging duration of 2 h (Fig. 1B). In comparison, no new distinct hypointense signal was detected on the luminal side in (1) HSS region (R4, within cuff) or (2) OSS region (R5, distal to cuff) of RCCA, and (3) throughout LCCA in the post-contrast images (Fig. 1B). In the IgG-MPIO control group, no new distinct hypointense signal was detected throughout RCCA and LCCA in post-contrast images (Online Resource Fig. 1).

**Fig. 2:**
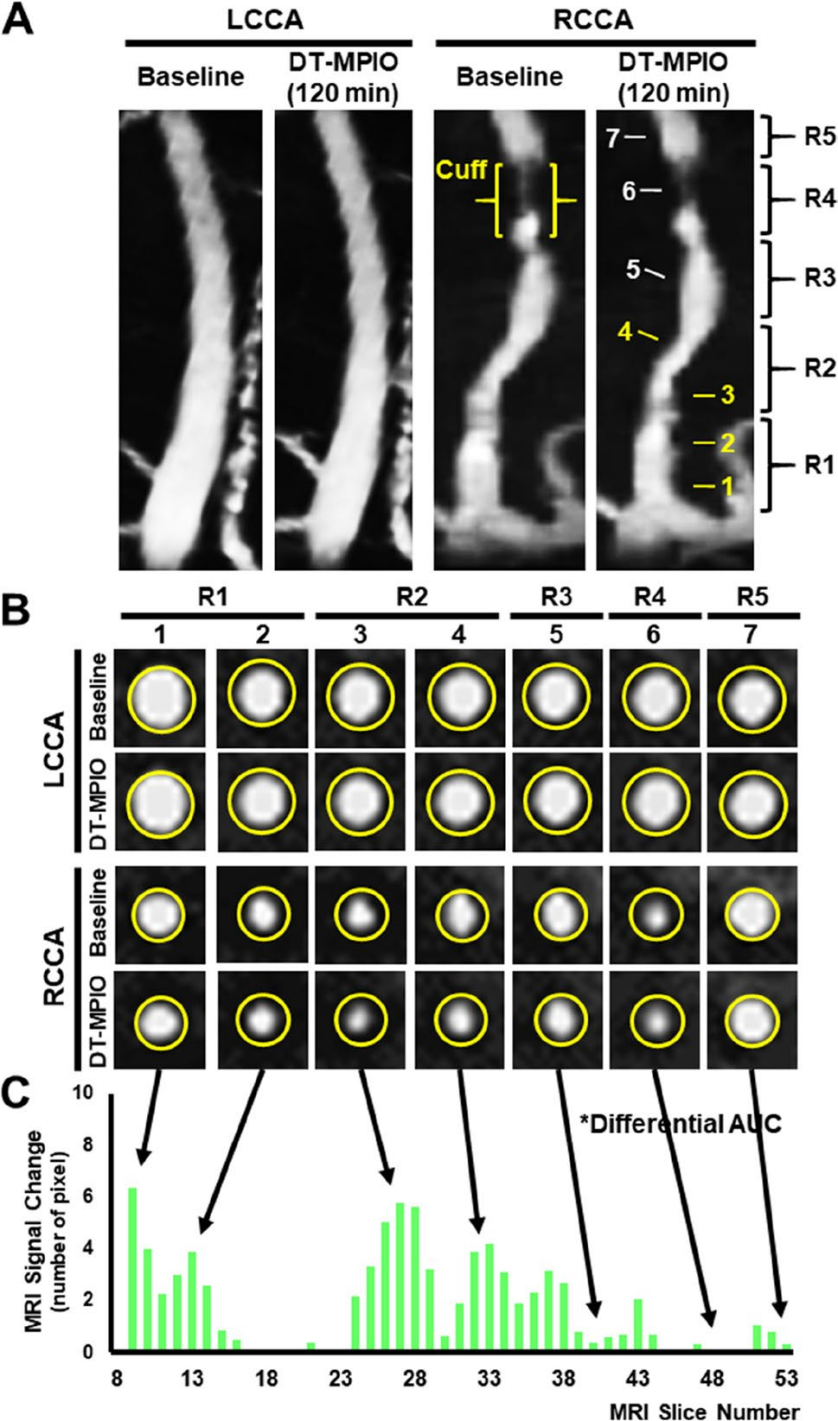
Quantification of magnitude of MR signal change. (A) The pre- and post-contrast 3D-TOF MRA images of RCCA and LCCA. (B) Axial MR images of RCCA and LCCA. New areas of hypointense signal were identified on the respective axial slices in R1–3 of RCCA. No new hypointense signal was detected on the axial slices in R4 and R5 of RCCA, and throughout LCCA in the post-contrast images. (C) Quantification of magnitude of MR signal change between the pre- and post-contrast carotid images. The magnitude of signal change was greater in R1–3 of RCCA than R4 or R5

**Fig. 3:**
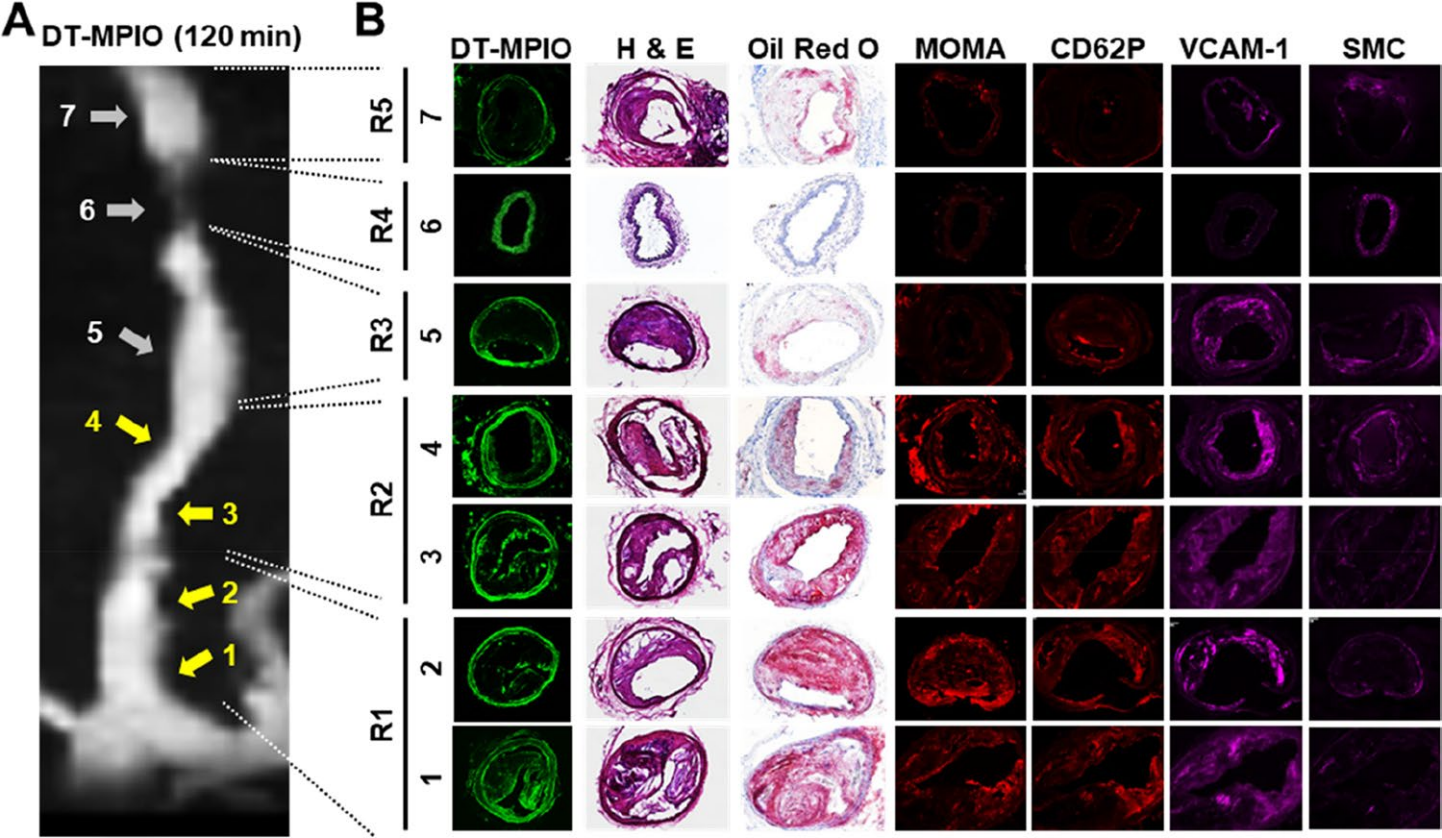
Histological analysis of all regions in RCCA. (A) The post-contrast (DT-MPIO) MRA image of the right common carotid artery (RCCA). The distinct hypointense signal in R1 and R2 was due to DT-MPIO targeting at the vulnerable inflamed plaques in the matching sections. No new hypointense signal was observed in R4 and R5, consistent with minimal or absent DT-MPIO binding to the disease free areas in R4 and the lesions of relatively stable phenotype in R5. (B) Histological analysis of all matching regions in the right common carotid artery. Matching histological sections showed that plaques in R1 and R2 have characteristics of a vulnerable inflamed plaque phenotype (i.e. high expression of inflammatory biomarkers: VCAM-1, P-selectin, MOMA, high lipid content and thin layers of smooth muscle cells (SMCs) in the cap of the plaque). Copious amount of fluorescent-labelled DT-MPIO was detected in plaques with a vulnerable inflamed phenotype in R1 and R2 of RCCA. Plaques were not observed in R4. No DT-MPIO was detected in the disease free areas in R4. Plaques in the R5 have characteristics of a relatively stable and less inflamed phenotype (i.e. low expression of inflammatory biomarkers: VCAM-1, P-selectin, MOMA, low lipid content and denser layers of SMCs). Insignificant amount of DT-MPIO was detected in these stable plaques in R5

Consistent with the 3D-TOF angiographic images (Fig. 2A), new areas of hypointense signal were identified on the respective axial slices in R1–3 of RCCA in the DT-MPIO group (Fig. 2B). In comparison, no new hypointense signal was detected on the axial slices in (1) R4 or (2) R5 of RCCA, and (3) throughout LCCA in the post-contrast images (Fig. 2B). Furthermore, the magnitude of signal change was greater in R1–3 region of RCCA than (1) R4 or (2) R5 (Fig. 2C), and (3) throughout LCCA in the post-contrast images (Online Resource Fig. 2).

#### Histological Validation

Histological examination confirmed that plaques were formed in R1–3 and R5 of RCCA post-cuff implantation in all animals (Fig. 3). However, the plaques had a remarkably different morphology. Plaques in the R1 and R2 have characteristics of a vulnerable inflamed plaque phenotype (i.e. high expression of inflammatory biomarkers: VCAM-1, P-selectin, MOMA, high lipid content and thin layers of smooth muscle cells (SMCs) in the cap of the plaque). Plaques in R5 have characteristics of a relatively stable and less inflamed phenotype (i.e. low expression of inflammatory biomarkers: VCAM-1, P-selectin, MOMA, low lipid content and denser layers of SMCs evenly dispersed in the intima). Plaques were not observed in R4 of RCCA (Fig. 3) or in control LCCA (Online Resource Fig. 2).

In the DT-MPIO group, majority of the fluorescent-labelled DT-MPIO was detected in plaques with a vulnerable inflamed phenotype in R1 and R2 of RCCA. Insignificant amount of DT-MPIO was detected in the plaques with a relatively stable and less inflamed phenotype in R5. The lack of DT-MPIO was verified in the disease free areas in R4 of RCCA (Fig. 3) and control LCCA (Online Resource Fig. 2). The findings proposed that DT-MPIO specifically target at lesions with vulnerable inflamed plaque phenotype, differentiating them from the stable, less inflamed plaques. Furthermore, the findings corroborated that the distinct hypointense signal observed on the post-contrast MRA in R1 and R2 of RCCA (Figs. 2 and 3) was due to DT-MPIO targeting at the vulnerable inflamed plaques in the matching sections (Fig. 3). The minimal or absent DT-MPIO in the histologic sections were consistent with the lack of distinct hypointense signal in the corresponding post-contrast MRA in the following: (1) the plaques of relatively stable phenotype in R5, (2) the disease free areas in R4 of RCCA (Fig. 3) and (3) the control LCCA (Online Resource Fig. 2). In the control group, no non-specific IgG-MPIO binding was detected throughout both carotid arteries (Online Resource Fig. 1). The findings were consistent with the absence of new distinct hypointense signal in RCCA and LCCA in the corresponding post-contrast MRA.

### Quantitative Reporting of the Inflammatory Status of Plaques for Risk Stratification

#### Stratifying the Risk of Plaques in Different Regions of RCCA

**Fig. 4:**
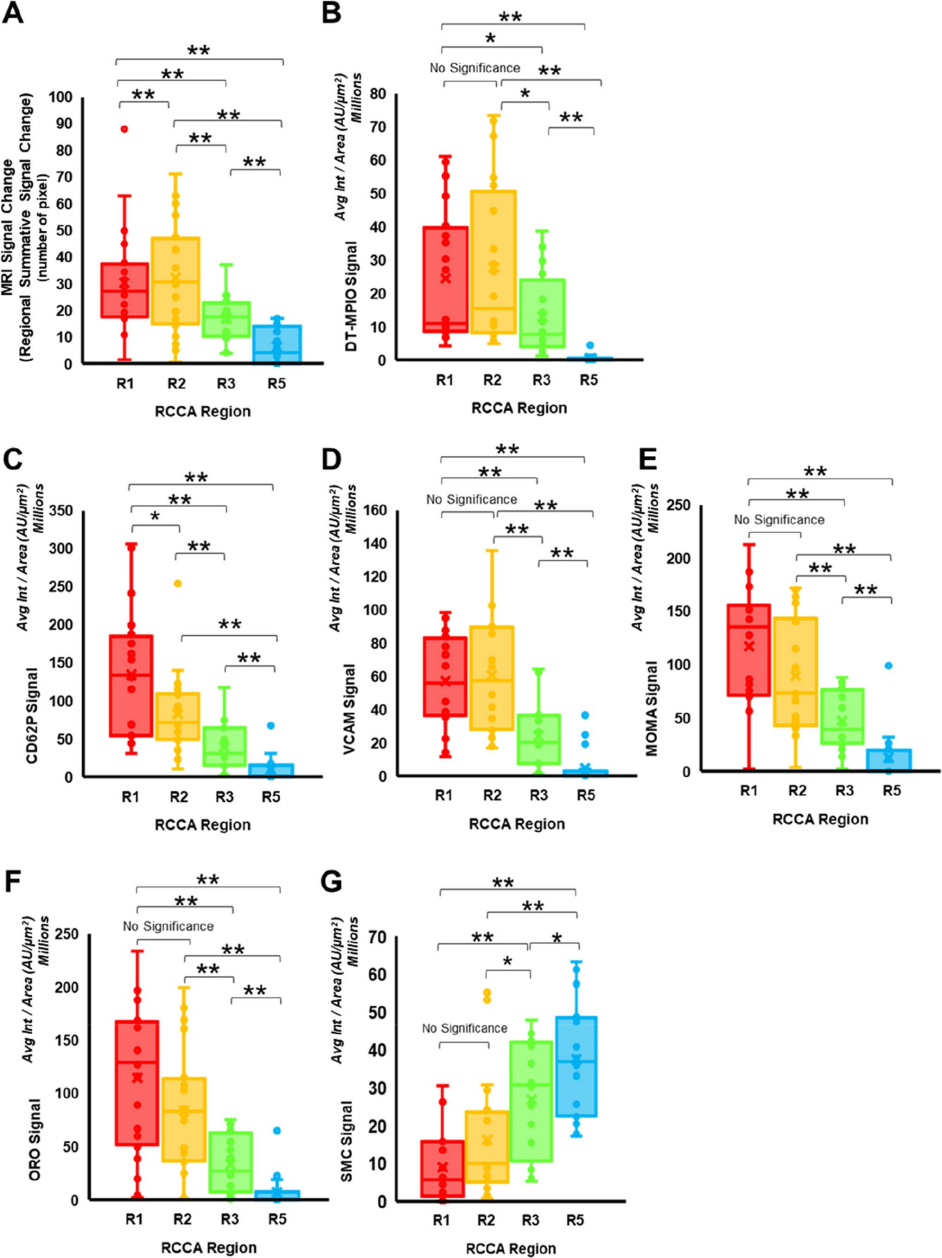
Stratifying the risk of plaques in different regions of RCCA. (A) MRI signal change (regional summative signal change), (B) DT-MPIO binding, (C) expression of P-selectin, (D) expression of VCAM-1, (E) expression of MOMA, (F) lipid and (G) SMC content in the plaques formed in different regions of the RCCA in all 20 animals were evaluated. Avg Int, average intensity; AU, fluorescence arbitrary unit

Furthermore, we examined whether DT-MPIO-enhanced MRI can quantitatively report on the inflammatory and vulnerability status of the plaques for risk stratification. In the DT-MPIO group, new areas of distinct hypointense signal were more conspicuous in R1 and R2 than that in R3. Absent or minimal new hypointense signal was detected in R5 (Fig. 2A and B). This was confirmed by significantly greater magnitude of MR signal change in R1 and R2 than that in R3 (*P* < 0.01), which was in turn greater than that in R5 (*P* < 0.01) (Figs. 2C and 4A). This gradation of signal change in R1–5 concurred with the matching histological results (Figs. 3 and 4): The plaques formed in R1 and R2 demonstrated the highest level of inflammation and their expression of P-selectin, VCAM-1 and MOMA was significantly higher than those in R3 (Fig. 4C–E, P-selectin: *P* < 0.01; VCAM-1: *P* < 0.01; MOMA: *P* < 0.01), which were in turn higher than those in R5 (Fig. 4C–E, P-selectin: *P* < 0.01; VCAM-1: *P* < 0.01; MOMA: *P* < 0.01). Similarly, the plaques in R1 and R2 also exhibited the highest level of vulnerability status (i.e. the highest lipid content, the lowest ‘stabilising’ SMC content) compared with those in R3, which were in turn more vulnerable and unstable than those in R5. The lipid content in the plaques formed in R1 and R2 was significantly higher than that in R3 (Fig. 4F, P < 0.01), which was in turn higher than that in R5 (*P* < 0.01). Conversely, the plaques in R1 and R2 bore the least ‘stabilising’ SMC content, which was significantly lower than that in R3 (Fig. 4G, P < 0.05), which was in turn lower than that in R5 (Fig. 4G, P < 0.05). Based on the inflammatory and vulnerability status, plaques formed in R1 and R2 were the highest risk vulnerable plaques whereas plaques in R3 and R5 were intermediate-risk plaques and low-risk stable plaques respectively. The high-risk plaques in R1 and R2 were targeted by significantly more substantial amount of DT-MPIO than the intermediate-risk plaques in R3 (*P* < 0.05), which were in turn more than the low-risk plaques in R5 (*P* < 0.01) (Fig. 4B).

The histological data corroborated that (i) the most prominent DT-MPIO-induced MR signal change was observed and quantified in high-risk plaques in R1 and R2; (ii) limited DT-MPIO-induced MR signal change was observed and quantified in intermediate-risk plaques in R3; and (iii) minimal DT-MPIO-induced MR signal change was observed and quantified in the low-risk stable plaques in R5. Hence, these results supported that DT-MPIO-enhanced MRI can quantitatively track the inflammatory and vulnerability status of the plaques in different regions of RCCA.

#### Stratifying the Risk of Individual Plaques Along RCCA

**Fig. 5:**
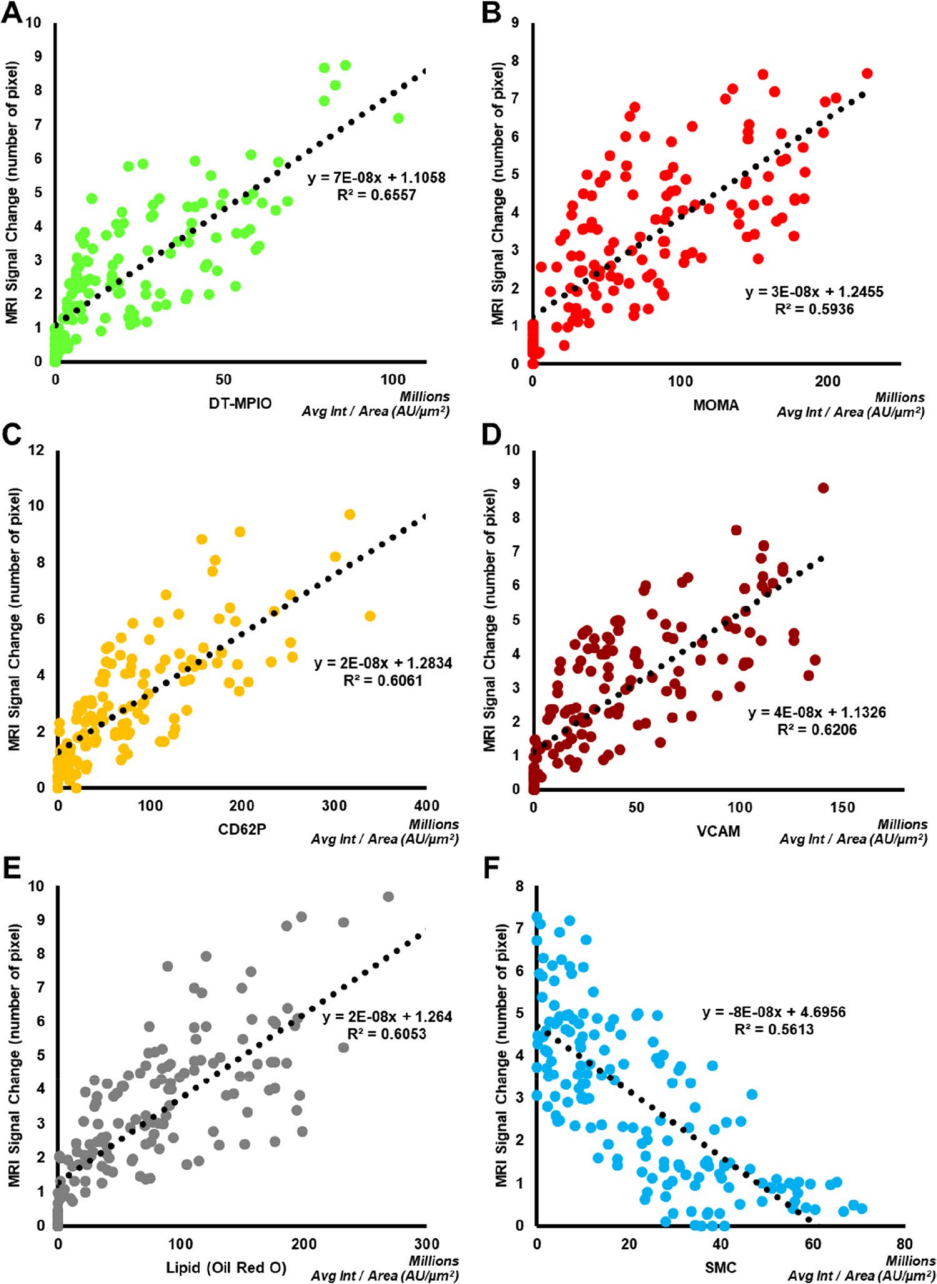
Quantitative reporting of the inflammatory status of individual plaques for risk stratification. The correlations between the magnitude of MR signal change induced by DT-MPIO, and the inflammatory and vulnerability status of individual plaques in all 20 mice were evaluated. (A) DT-MPIO signal. (B) MOMA expression (macrophage burden). (C) P-selectin expression. (D) VCAM-1 expression. (E) Oil Red O (lipid content). (F) Smooth muscle cell content. Avg Int, average intensity; AU, fluorescence arbitrary unit

Finally, we examined the correlations between the magnitude of MR signal change induced by DT-MPIO, and the inflammatory and vulnerability status of individual plaques in all 20 mice. Firstly, magnitude of MR signal change was significantly correlated with the amount of DT-MPIO targeting at the plaques (*R*^2^ = 0.66, *P* < 0.01) (Fig. 5A). Moreover, significant correlation between magnitude of MR signal change and the expression of all inflammatory biomarkers was demonstrated (i.e. MOMA-1 (*R*^2^ = 0.59, *P* < 0.01), CD62P (*R*^2^ = 0.61, *P* < 0.01), VCAM-1 (*R*^2^ = 0.62, *P* < 0.01); Fig. 5B–D). Furthermore, magnitude of MR signal change was significantly correlated with plaque vulnerability (i.e. significantly correlated with lipid content (Fig. 5E, R^2^ = 0.61, *P* < 0.01) and inversely correlated with smooth muscle cell content, one of the ‘stabilising’ components in the plaque (Fig. 5F, R^2^ = 0.56, *P* < 0.01)). These results corroborated that DT-MPIO-enhanced MRI can stratify the risk of individual plaques by quantitatively reporting their inflammatory and vulnerability status.

## Discussion

The management of asymptomatic carotid disease remains perpetually contentious. The most controversial issue is the growing body of evidence that the stroke risk among medically treated asymptomatic patients has reduced across all stenosis severities, attributable to advances in medical therapy, such as statins, antiplatelets and, more recently, dual pathway inhibition with low-dose rivaroxaban [17, 18]. It is now suggested that the annual stroke risk may fall to 0.5–1% [3] — a rate that is comparable to that seen in Asymptomatic Carotid Trial (ACT I) and Carotid Revascularization Endarterectomy versus Stenting Trial (CREST) after successful stenting or endarterectomy [19, 20]. Moreover, 94% of carotid interventions in the USA are of limited utility, costing $2.1 billion per year, only prevent 1–4% of all strokes and placing patients at risk of periprocedural stroke [3]. Yet, there are approximately 10 to 15% of patients with asymptomatic carotid plaques are still at a higher risk for stroke, despite best medical therapy [21]. This clinical subgroup is most likely to benefit from carotid intervention. The current challenge is to accurately identify and target only these high-risk asymptomatic individuals with prophylactic carotid intervention, leaving the mass of lower risk patients to be treated with medical therapy only [3, 21].

Wide range of promising non-invasive imaging techniques have been used to interrogate plaque vulnerability and inflammation in vivo, adding further prognostic information to luminal stenosis alone.^18^F-FDG PET imaging has offered useful insights on inflammatory dynamics in the plaques, and is a promising clinical tool to predict early stroke recurrence and monitor anti-inflammatory effects of atheroma pharmacotherapies [22–24]. However, radiation exposure and requirement of specialist facilities associated with PET (and co-registered CT) may impose limitations on the routine use of this imaging modality in clinical assessment of atherosclerotic disease [25]. Contrast-enhanced ultrasound (CEUS) demonstrated that microbubbles, as intravascular tracers, were retained in areas of plaque inflammation and angiogenesis in human carotid atherosclerosis [26]. Through the use of targeted gas-filled microbubbles, it has been possible to image a range of targets, including VCAM-1 [27] and P-selectin [28], in the pre-clinical studies. CEUS may provide valuable information for further risk stratification of echolucent plaques and carotid artery stenosis, beyond that provided by standard ultrasound imaging. Carotid MRI has significantly advanced the field of atherosclerosis imaging. The ability of in vivo carotid MRI to identify and quantify the main plaque components such as intraplaque haemorrhage (IPH), lipid-rich necrotic core and surface disruption has been meticulously validated with histology [29]. In particular, carotid MRI detection of IPH is consistently associated with future cerebrovascular events [30]. Despite the marked advance in structural and functional plaque MRI techniques, direct reporting of the specific inflammatory activities within the plaques was limited until the application of USPIO [7, 8] and MPIO [9–11].

Herein, we have developed DT-MPIO enhanced MR imaging tool to (i) target and differentiate vulnerable carotid plaques from stable plaques; and (ii) quantitatively report the inflammatory status of plaques for risk stratification. Exploiting the rapid binding at molecular targets and fast blood clearance of unbound DT-MPIO, this molecular imaging tool can attain prominent and quantifiable signal effects in vivo.

Nanoscale particles, such as USPIOs, were utilised in clinical setting as macrophage imaging agents to track plaque inflammation for risk stratification. Uptake of USPIO was shown in both symptomatic and contralateral asymptomatic carotid artery in patients with carotid atherosclerosis [31]. The ATHEROMA study (Atorvastatin Therapy: Effects on Reduction of Macrophage Activity) demonstrated that high-dose statin over a 3-month period has attenuated plaque inflammation with substantial reduction in USPIO uptake in the human carotid arteries [8]. However, the prolonged blood half-life of USPIO increases the blood pool contrast effects. This results in long lag time between USPIO administration and imaging, limiting the use in acute clinical events on a large scale. The interval of USPIO circulation time in most animal models and patients was 2 to 4 days [32, 33], and 24 to 36 h, respectively [8, 34], although the targeted USPIO reduced this period to between 8 and 24 h in animal studies [35, 36]. Furthermore, the intraplaque macrophages are dynamic with transient residence within lesions [37]. In the acute clinical events, such as stroke, the prolonged imaging interval may pose limitations to discriminate if the signal detection was due to plaque destabilisation that caused symptoms or the aftermath of clinical event.

In comparison with USPIO, ligand-conjugated MPIO may be more advantageous for imaging acute inflammatory processes in diseases such as atherosclerosis [9–11] and ischaemic-reperfusion injury [38]. This work further demonstrated the benefits of DT-MPIO in imaging acute clinical conditions: (i) The higher iron load of MPIO significantly augments the sensitivity to attain in vivo detection and quantification of molecular targets. (ii) The half-life of our DT-MPIO (1.75 min) was substantially shorter than that of USPIO (11 h) [9, 35] The fast blood clearance of unbound DT-MPIO attenuates the blood pool contrast effect, augmenting the target-to-background ratio, resulting in conspicuous signal detected in the plaques (Figs. 1, 2 and 3). (iii) DT-MPIO against P-selectin and VCAM-1 are synthesised to simulate the swift binding of monocytes in the circulation to the artery wall and have shown synergistically augmented binding effect to both adhesion molecules [9–11]. Due to the rapid antigen–antibody response, prominent DT-MPIO-induced MR signals were identified as early as 30 min and lasted up to 2 h after injection (Fig. 1); a practical imaging interval for acute thromboembolic events.

Patient symptomatology and the luminal stenosis assessed by conventional angiographic techniques are no longer appropriate to be the sole selection criteria for surgical intervention. The Asymptomatic Carotid Surgery Trial (ACST) showed that there is a subgroup of high-risk asymptomatic patients who bear the vulnerable plaques, which cannot be detected by the conventional angiographic techniques [39] Building on previous studies [9, 15, 16], we have used the peri-arterial cuff to generate plaques with varying degree of (i) phenotypes (from stable to unstable vulnerable phenotypes), (ii) inflammation levels (from low, moderate to high inflammation) and (iii) risk levels (from low, intermediate to high-risk), at specific anatomical locations along the same single carotid artery. We then utilised this valuable platform to test the targeting capabilities of DT-MPIO to (i) differentiate the high-risk plaques from the stable ones and (ii) quantitatively report the inflammatory status of the plaques in the same single artery, eliminating the local haemodynamic variation and animal-to-animal variation. Herein, we demonstrated that in vivo DT-MPIO-enhanced MRI can (i) target high-risk vulnerable inflamed plaques and (ii) differentiate the heterogeneity (i.e. high-risk vulnerable plaques in R1 and R2 vs intermediate-risk plaques in R3 vs low-risk stable plaques in R5) within the asymptomatic plaque population in mouse carotid artery within a practical imaging interval.

It is noteworthy that low-grade luminal stenosis on the baseline MRA (Figs. 1 and 2) has underestimated the severity of atherosclerotic disease burden due to expansive vascular remodelling. This highlighted that the vulnerable plaques could be missed by conventional MRA. However, these vulnerable plaques were identified and characterised by DT-MPIO-enhanced MRA (Figs. 1, 2 and 3). This molecular imaging tool may surmount the limitations of existing angiographic techniques to look beyond lumen stenosis by directly reporting the intraplaque inflammation. The plaques were generated in the carotid artery, advancing a step closer to translate this in vivo molecular imaging tool to patients with carotid artery disease.

Quantitative reporting of intraplaque inflammatory activity is the main goal in characterisation and risk stratification of carotid atherosclerosis. Herein, we have demonstrated that DT-MPIO-enhanced MRI can quantitatively report and closely track the inflammatory activity and vulnerability status of the local plaques. Through this, the molecular imaging tool can stratify the risk of plaques at specific regions of the carotid artery (i.e. conspicuous MR signal detected in high-risk plaques in R1 and R2, moderate signal in intermediate-risk plaques in R3 and minimal signal in low-risk plaques in R5). This concurred with our earlier studies that characterised VCAM-1 and selectins on (i) activated endothelial cells [40], (ii) human carotid plaques [14] and (iii) plaques in aortic root and carotid artery in murine model [9] by MRI using antibody-conjugated iron particles. Our study showed that the DT-MPIO-enhanced MRI might allow characterisation of plaque vulnerability and inflammation across a range of atherosclerotic lesions complexities in the future. The significant contrast sensitivity, swift binding and blood clearance of DT-MPIO enhance a conspicuous ‘target-to-background’ and quantifiable signal effect within a practical imaging interval. These features distinguish DT-MPIO-enhanced MRI as an invaluable molecular imaging tool in imaging acute thromboembolic events. With further clinical translation, these attributes would be ideal for clinical application.

Similar to our previous study [9], animals displayed no adverse effect after injection of DT-MPIO. Iron oxide particles, such as Ferumoxytol, have demonstrated a good safety profile and have been clinically approved as an intravenous iron replacement therapy in patients with iron deficiency anaemia [41]. Iron oxide particles were also used as MRI contrast reagent in clinical vascular and atherosclerosis imaging [8, 42, 43], and offer an attractive option for 20–40% vascular patients with chronic kidney disease, which could prevent the administration of conventional iodine- and gadolinium-based contrast agents [42, 43].

The amount of MPIO used in this study was 30 mg iron/kg body weight. This dose was higher when compared to non-targeted iron-based contrast agents for human atherosclerosis imaging (3–4 mg/kg) [42, 43], but lower than USPIO-based pre-clinical imaging studies involving mice and rabbits (10–56 mg/kg) [7, 33, 35, 36]. The study selected a moderately high dose to allow quantitative reporting of the local plaque inflammation for risk stratification of carotid atherosclerosis, and would require additional optimisation in future studies. Although conspicuous hypointense signal was attained in this study, further development and exploration of other imaging sequences, such as susceptibility weighted sequence, can augment the sensitivity for active molecular imaging in vivo. Future studies will involve validation of this molecular imaging tool in large animal models.

The ESVS guidelines are the first clinical guidelines that incorporate imaging features in the management of asymptomatic carotid disease. This molecular MRI tool can potentially allow us to move beyond luminal stenosis to characterise plaque vulnerability and quantitatively report the inflammatory activity in atherosclerosis, further refining risk stratification of asymptomatic patients for stroke prevention. This facilitates accurate identification and targeting only the high-risk asymptomatic individuals with prophylactic carotid intervention, whilst the majority of lower risk patients to be treated medically. With further clinical translation, this molecular imaging tool could potentially pave the way for personalised management of carotid atherosclerotic disease.

## Supplementary Information

Below is the link to the electronic supplementary material.

Supplementary file1 (DOCX 1333 KB)

## Data Availability

The data that support the findings of this study are available from the corresponding author upon reasonable request.

## Abbreviations

ACST: Asymptomatic Carotid Surgery Trial
ACT I: Asymptomatic Carotid Trial
ApoE^−^^/^^−^: Apolipoprotein E-deficient mice, ApoE knock-out mice
ATHEROMA study: Atrovastatin Therapy: Effects on Reduction of Macrophage Activity
AUC: Area under the curve
CD62P: P-selectin
CREST: Carotid Revascularization Endarterectomy versus Stenting Trial
DT-MPIO: Dual-targetting microparticles of iron oxide
ESVS: European Society for Vascular Surgery
FLASH: Fast low-angle shot
HSS: High shear stress
LCCA: Left common carotid artery
LSS: Low shear stress
MOMA-2: Anti-macrophages/monocytes antibody
MRA: Magnetic resonance angiography
MRI: Magnetic resonance imaging
ORO: Oil Red O
OSS: Oscillatory shear stress
RCCA: Right common carotid artery
SMC: Smooth muscle cell
TOF MRA: Time-of-flight magnetic resonance angiography
USPIO: Ultrasmall superparamagnetic iron oxide
VCAM-1: Vascular cell adhesion molecule-1
