# Supplementary material for: Development of Molecular Magnetic Resonance Imaging Tools for Risk Stratification of Carotid Atherosclerotic Disease Using Dual-Targeted Microparticles of Iron Oxide
Source: Transl Stroke Res. 2021 Jul 25;13(2):245–56. doi: 10.1007/s12975-021-00931-3 (PMC8918460; doi:10.1007/s12975-021-00931-3)
Supplement: Supplementary file 1 — Supplementary file1 (DOCX 1333 KB) [file 12975_2021_931_MOESM1_ESM.docx]

**Supplementary Information for**

**Development of molecular magnetic resonance imaging tools for risk stratification of carotid atherosclerotic disease using dual-targeted microparticles of iron oxide**

**AUTHORS:**

^*^Joyce MS Chan MBBS PhD^1,2^, Park Sung Jin PhD^1^, Michael Ng BSc^1^, Joanne Garnell BSc^1^, Chan Wan Ying MBBS FRCR^3^, Chong Tze Tec MBBS FACS^2^, Kishore Bhakoo PhD^4^

^1^Translational Cardiovascular Imaging Group, Institute of Bioengineering and Bioimaging (IBB), Agency for Science, Technology and Research (A*STAR), Singapore

^2^Department of Vascular Surgery, Singapore General Hospital, SingHealth, Singapore

^3^Division of Oncologic Imaging, National Cancer Centre, SingHealth, Singapore

^4^Institute of Bioengineering and Bioimaging (IBB), Agency for Science, Technology and Research (A*STAR), Singapore

***CORRESPONDING AUTHOR:** Joyce M. S. Chan

^1^Translational Cardiovascular Imaging Group, Institute of Bioengineering and Bioimaging (IBB), Agency for Science, Technology and Research (A*STAR), Singapore

^2^Department of Vascular Surgery, Singapore General Hospital, SingHealth, Singapore

Email: joyce_chan@ibb.a-star.edu.sg

**Contents of this SI file include:**

- Supplementary Methods
- Supplementary Table 1 and 2
- Supplementary Figure 1 and 2

**List of Supplementary Methods:**

1. **Supplementary Table 1**. Primary antibody information
2. **Supplementary Table 2**. Secondary antibody information

**List of Supplementary Figures:**

1. **Supplementary Figure 1.** Control: In vivo MRI of mouse carotid arteries using IgG-MPIO.
2. **Supplementary Figure 2.** Control: In vivo MRI of LCCA using DT-MPIO.

**Supplementary Methods**

**Synthesis of fluorescent-tagged dual antibody-conjugated MPIO**

Dual-targeted MPIO was prepared by incubating iron oxide micro-particles (1μm diameter; Dynabeads™ MyOne™ Tosylactivated; Invitrogen, Singapore) with fluorescein cadaverine (LifeTech, Singapore), purified monoclonal rat anti-mouse antibodies for VCAM-1 (CD106; BD Pharmingen™; Zuellig Pharma, Singapore) and P-selectin (CD62P; Santa Cruz Biotechnology; Axil Scientific, Singapore) together at 37°C, 20 hours, with continuous rotation (20 mg MPIO per 40 µg of each antibody). Control MPIO was prepared in the same way with fluorescein cadaverine and purified monoclonal rat anti-mouse antibodies for IgG-1 (AbD Serotec; SciMed, Singapore) (20 mg MPIO per 80 µg antibody). Post incubation, MPIO was rinsed twice in wash/store buffer (PBS pH 7.4, 0.1% bovine serum albumin (BSA), 0.05% Tween® 20) at 4°C and incubated with blocking buffer (PBS pH 7.4, 0.5% BSA, 0.05% Tween® 20) overnight at 37°C, to block unreacted tosyl sites. Finally, MPIO was washed twice with wash/store buffer at 4°C, 5 minutes, and kept at 4°C at 40 mg iron per ml PBS (0.1% BSA).

**Histology and immunohistochemical staining of carotid arteries**

**Tissue sample preparation**

ApoE-/- mice were euthanized at the end of MRI scan for histological assessments. Both RCCA and LCCA of each animal were surgically removed under stereomicroscope and fixed with 4% paraformaldehyde in phosphate buffered saline (PBS) at 4°C for 2 hours. The samples were washed with PBS and left in 30% sucrose in PBS to dehydrate the tissue. After which, the samples were frozen with optimal cutting temperature (OCT) compound and serially sectioned at 12 µm thickness on a cryostat (Leica CM1950®) for further studies.

**Haematoxylin and eosin (H & E) stain**

The sectioned samples were washed with PBS to remove OCT compounds embedded around the tissue. Haematoxylin solution Gill No.3 (Sigma-Aldrich®, GHS316) was added to incubate the samples for 5 minutes before washing with distilled water for 10 minutes to induce metachromatic stain. The samples were then treated with Eosin Y solution (Sigma-Aldrich®, HT10132) for 1 minute to stain the cytoplasm of the cells and briefly rinsed with distilled water. The samples were treated with 70, 80, 90, 100% ethanol and 100% xylene for dehydration, and mounted with a cover glass and Canada Balsam mounting media.

**Oil red O stain**

Frozen-sectioned samples were washed with distilled water and rinsed with 60% isopropanol. Freshly prepared Oil red O (ORO) solution (final concentration: 0.3 g ORO, 60mL isopropanol, 40mL distilled water) was applied for 15 minutes after which the samples were briefly washed with 60% isopropanol. The samples were then washed with PBS and mounted with ProLong^TM^ Gold Antifade Mountant (Thermo Fisher Scientific).

**Immunohistochemistry**

The sectioned samples were rinsed with PBS to remove OCT compound and treated with 1% bovine serum albumin for 30 minutes to block non-specific binding. Dual primary antibodies with different hosts (Supplementary Table 1) were then applied. Samples were maintained overnight at 4°C. The next day, samples were washed with PBS, after which, the samples were treated with secondary antibodies (Supplementary Table 2) for 2 hours at room temperature, to obtain a different colour for each protein marker. The samples were rinsed with PBS, mounted with ProLong^TM^ Gold Antifade Mountant with DAPI (ThermoFisher Scientific) and observed under fluorescent or confocal microscope.

**Supplementary Table 1. Primary antibody information.**

|  | **Primary antibody** | **Host** | **Company** | **Cat. No.** | **Dilution factor** |
| --- | --- | --- | --- | --- | --- |
| **4 basic antibodies** | Monocyte + macrophage  (MOMA-2) | Rat | Abcam | Ab33451 | 1:100 |
|  | Alpha smooth muscle actin | Goat | Abcam | Ab21027 | 1:200 |
|  | CD62P (p-selectin) | Mouse | Abcam | Ab54427 | 1:100 |
|  | Vascular cell adhesion protein 1  (VCAM-1) | Rabbit | Abcam | Ab134047 | 1:100 |

**Supplementary Table 2. Secondary antibody information.**

|  | **Secondary antibody** | **Host** | **Company** | **Cat. No.** | **Dilution factor** |
| --- | --- | --- | --- | --- | --- |
| **Fluorescent conjugated** | AlexaFluore^®^ 594 conjugated Rat IgG H&L | Donkey | Abcam | Ab150156 | 1:4000 |
|  | AlexaFluore^®^ 647 conjugated Goat IgG H&L | Donkey | Abcam | Ab150135 | 1:4000 |
|  | AlexaFluore^®^ 594 conjugated Mouse IgG H&L | Donkey | Abcam | Ab150112 | 1:5000 |
|  | AlexaFluore^®^ 647 conjugated Rabbit IgG H&L | Donkey | Abcam | Ab150063 | 1:4000 |

**Supplementary Results**


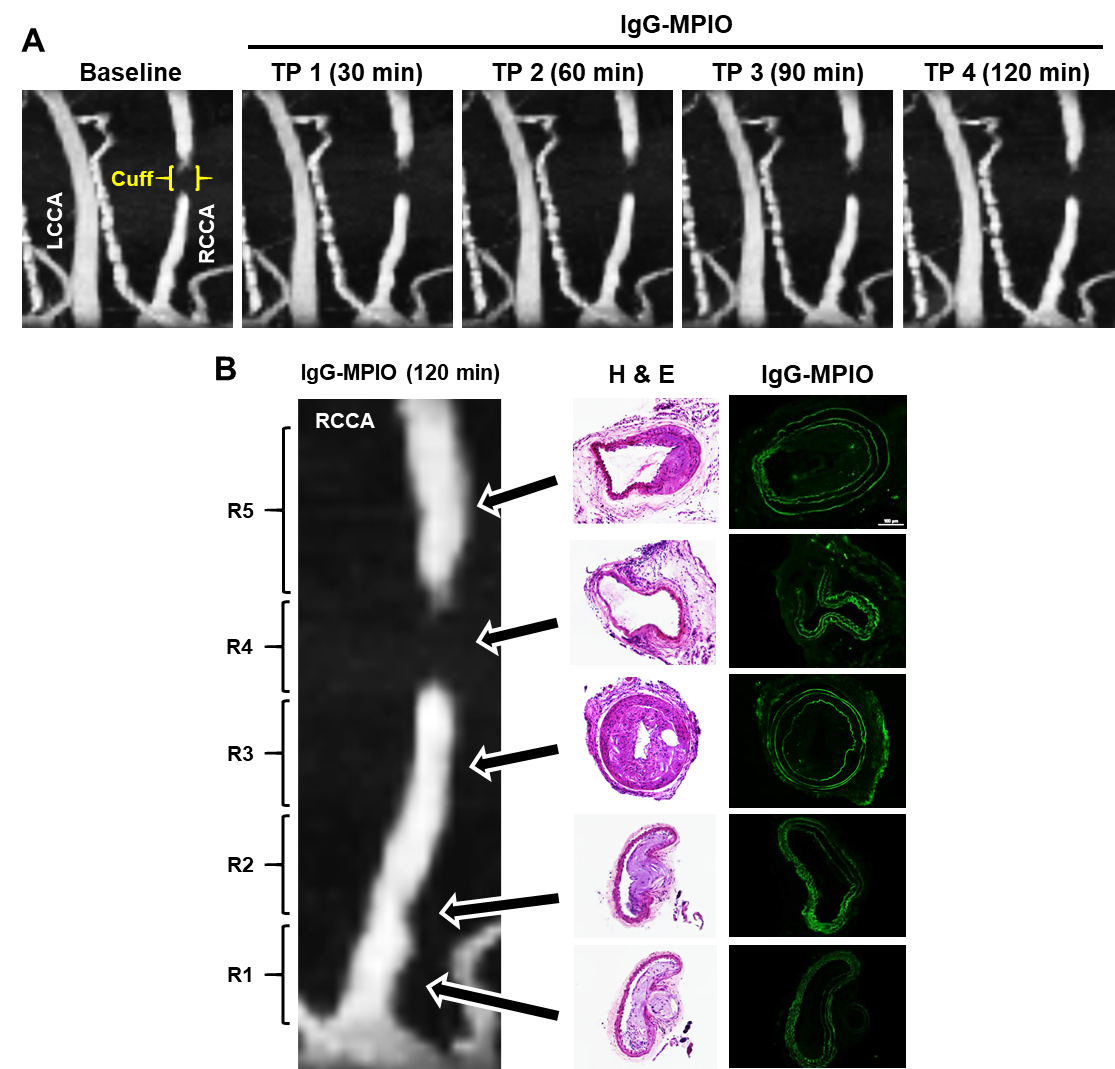


**Supplementary Figure 1. Control: *In vivo* MRI of mouse carotid arteries using IgG-MPIO. A. *In vivo* MRI in the IgG-MPIO control group**. No new distinct hypointense signal was detected in R1-5 of RCCA and throughout LCCA in post-contrast images for the entire 2-hour imaging period. **B. Histology of RCCA in the IgG-MPIO control group.** Matching histological sections revealed no non-specific IgG-MPIO binding to the plaques in R1-3 and R5 or athero-protective region in R4. The results were consistent with the absence of new distinct hypointense signal throughout R1-5 of RCCA in the matching postcontrast MRA.


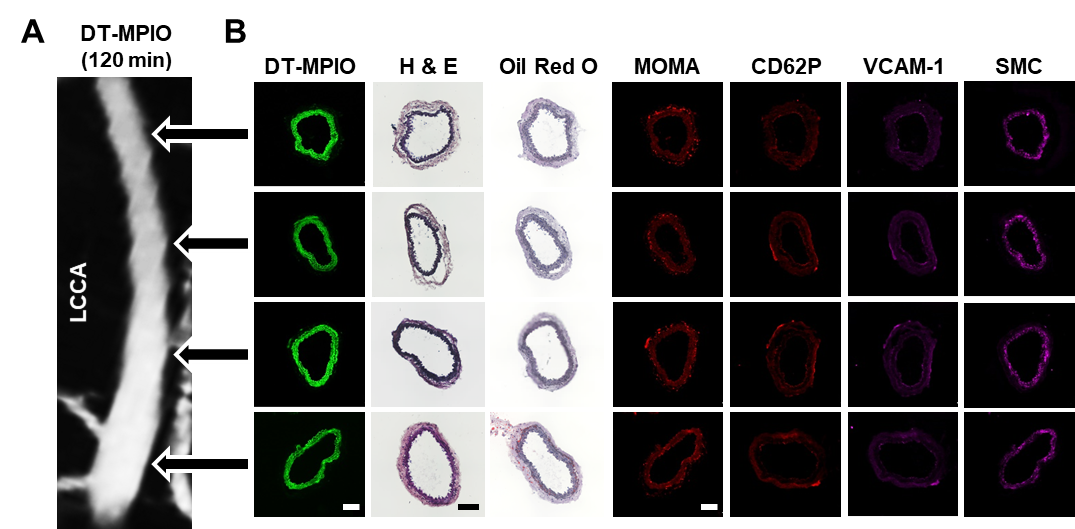


**Supplementary Figure 2. Control: *In vivo* MRI of LCCA using DT-MPIO. A. Post-contrast MRA image of control LCCA at 2 hours after DT-MPIO injection**. No new distinct hypointense signal was detected throughout LCCA in post-contrast MRA image. **B. Histology of control LCCA.** Matching histological sections revealed no atherosclerotic plaque was observed in the nontreated LCCA. Absence of DT-MPIO was confirmed in the disease free areas of LCCA.
